# The political economy of multilateral lending to European regions

**DOI:** 10.1007/s11558-020-09385-y

**Published:** 2020-07-02

**Authors:** Zareh Asatryan, Annika Havlik

**Affiliations:** 1 ZEW Mannheim, Mannheim, Germany; 2 CESifo, Munich, Germany; 3 University of Mannheim, Mannheim, Germany

**Keywords:** Political economy of international organizations, Regional favoritism, European Investment Bank, European Union, D72, F53, G2

## Abstract

We study the political economy of allocation decisions within a major state investment bank. Our focus is the European Investment Bank (EIB) – “The Bank of the EU” – which is the largest multilateral lending (and borrowing) institution in the world. We study the behavior of about 500 national representatives at the EIB’s Board of Directors – the bank’s decisive body for loan approvals – and show that a representative’s appointment increases the probability that the sub-national region where she works receives a loan by about 17 percentage points. This “home-bias” effect is driven by large loans financing infrastructure projects. We discuss several pieces of evidence, which are consistent with the hypothesis that this home-bias lending may be due to favoritism, however, we cannot conclusively demonstrate this case of resource misallocation.

**Electronic supplementary material:**

The online version of this article (10.1007/s11558-020-09385-y) contains supplementary material, which is available to authorized users.

## Introduction

We study the political economy of loan allocation decisions of the European Investment Bank (EIB) or “the bank of the European Union”. We ask whether these lending decisions made by the powerful Board of Directors of the EIB, which consists of representatives of European Union (EU) Member States, purely follow the economic and EU integration related goals set by the bank, or if they can be explained instead by political or personal motives of the Member State representatives running the bank. Beyond the direct importance of understanding what determines the lending of the world’s largest multilateral lending (and borrowing) institution, this work may provide additional insights into the question of how the interplay between the incentives of non-elected officials and national interests shapes the policies of international organizations.

**Fig. 1:**
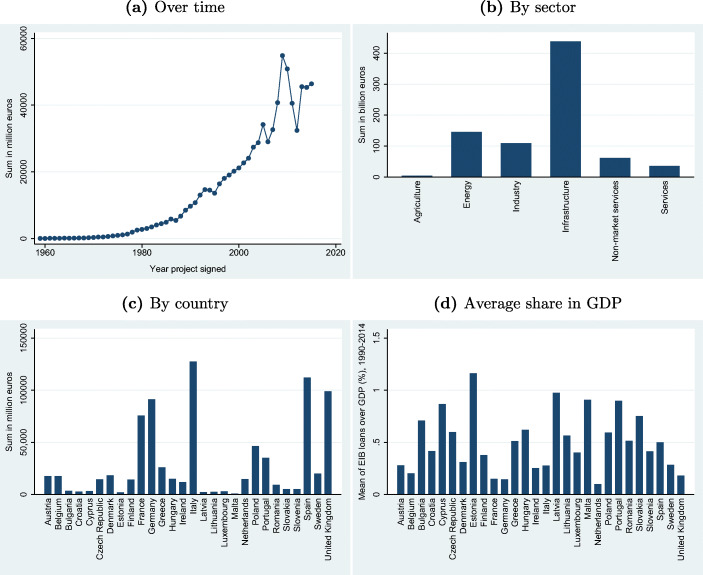
Descriptives on the size, evolution, and distribution of EIB loans. The figures are constructed from EIB data taken from EIB (2020c). Data on GDP is in 2005 prices and is taken from the European Regional Database

With the emergence of the European project, the EIB has gained importance with near exponential rates of growth in lending over the last few decades (see, Fig. 1a). In 2017, new commitments by the EIB in Europe summed up to around 76 billion Euros (EIB 2017b).^1^ The EIB together with the European Fund for Strategic Investments also serves as an important investment instrument in Europe, with current debates on the economic governance of the Eurozone often highlighting an even larger future role for the EIB (2015b, 2018).^2^

There are important reasons to believe that state investment banks like the EIB can serve the general interest of society. Traditional arguments stress the market failure fixing roles of these banks (Stiglitz 1994), while more recent views celebrate the capacity of these banks to invest in large and risky innovative projects, which may potentially have positive spillovers across the whole economy (Mazzucato and Penna 2016). The hopes of European policy makers to secure funding for large public investment projects, which the private markets fail to provide, are often tied to the EIB since the small EU budget typically cannot afford to finance these (Clifton et al. 2018). However, a potential trade-off is whether this type of government intervention is prone to other forms of governance failure such as rent-seeking by the technocrats running the bank. Since the EIB is not under democratic scrutiny directly, another hope is that this financing instrument would be largely free of the constraints of distributive politics such as pork barrel type of spending. This goes in contrast to the EU budget, which has too often served as a tool to please political and national appetites.^3^ On the other hand, the absence of electoral incentives and related constraints of political accountability may open other opportunities for decision makers at the EIB to discriminate in lending decisions such as based on their personal gains or preferences.

Our specific focus is the Board of Directors, which is the decisive body for the approval of loans at the EIB. This body includes a Director nominated by each EU Member State and one from the European Commission. We hand-collect data on the careers of the population of all 470 Directors with the aim of measuring the connections of Directors at the level of European sub-national regions as far as these can be captured by the Directors’ work experience.^4^ We then match the Director-level data to administrative project-level data of EIB loans granted since the foundation of the Bank in 1959^5^ again aggregated at the level of European regions. We describe the data, provide access to it, and explain the programs we use in the ??, which is posted on the website of the journal.

Using difference-in-differences and distributed lag models, we provide evidence supporting the hypothesis that lending is more likely to flow to the home regions of Directors compared to other regions upon appointment at the Board. This phenomenon, that we label “home-bias” effect, amounts to an average 17 percentage points (or 40% of the sample mean) increase in the likelihood of receiving a loan. Interestingly, the home-bias effect is entirely driven by a relatively small sub-set of very large infrastructural mega-projects.

One crucial question regarding the home-bias effect that we document is whether these discriminatory lending practices facilitate economic efficiency or whether they result in inefficient misallocation of resources. There are several potential explanations behind the home-bias effect, all of which predict a larger flow of transactions into the home regions,^6^ but with divergent predictions on the economic value of these transactions. On the one hand, Directors may have a personal gain in transferring resources to their home regions. The EIB’s rules of “Code of Conduct for the Members of the Board of Directors” EIB (2012) reveal the potential existence of issues of this nature by preventing former Board members to “lobby with members of the EIB governing bodies and Bank staff for their business, client or employer” within six months after leaving the Board. We label this mechanism as favoritism. The EIB is different from democratic contexts where politicians have electoral motives, however, in addition to personal gains due to favoritism, the directors may also simply have social preferences towards their home regions. For example, Transparency International EU (2016) points out that senior managers of the EIB have a lot of freedom to favor their home countries without citing the reasons to do so. Either way, this favoritism or preference based discrimination in lending practices will likely lead to resource misallocation. On the other hand, Directors may be able to reduce information asymmetries between the EIB and the borrowers in their region of work, thereby creating more value for both parties. For example, an informed Director may be able to reduce search costs or relax the costly needs of enforcement effort by identifying the set of projects most worthy of investments.

Five pieces of evidence speak against the information hypothesis. First, we study a sub-sample of Directors who change their work region during or after their service at the EIB. While sending money to their pre-EIB regions can be a mix of the information and favoritism channels, we show some evidence that the resources are sent to the post-EIB regions during the end of the Board membership, which is likely to be due to favoritism assuming that the Directors cannot have a priori information about the new region. Second, as laid out by Rajan (1992), Persson and Zhuravskaya (2016), and Fisman et al. (2017) the degree of information a Director has about a region (measured by her length of experience at the region) should be positively correlated with the amount of home-bias lending. Third, following (Cornell and Welch 1996; Fisman et al. 2017) we hypothesize that more informed lending practices should increase the variance of loan sizes because with more precise signals the Director’s prior beliefs of borrower quality have a wider distribution. Our evidence does not support either of these hypotheses. Fourth, our evidence that the home-bias phenomenon is entirely driven by infrastructural mega-projects may be more consistent with favoritism rather than the informational channels since it is likely that there is already much common information about such project as compared to smaller and more sophisticated projects. This finding is also in line with Do et al. (2017) and Persson and Zhuravskaya (2016) who show that favoritism in Vietnam and China, respectively, operates through expenditures on construction infrastructure rather than social expenditures such as on education. Finally, following (Persson and Zhuravskaya 2016), we study the timing of formation of the home-bias and show that the additional lending is flowing to regions of Directors’ workplace rather than their education regions, which may speak against the hypothesis that favoritism is driven by social preferences rather than by personal gain.

Overall, our evidence is consistent with the view that the regional home-bias at the EIB is driven by the favoritist practices of its Directors thus leading to resource misallocation and economic inefficiency. However, we ultimately fail to reject that other efficiency-enhancing factors can be responsible for the home bias effect.^7^ We also note that our findings are based on observable connections that are self-reported on CVs of Directors. Unobservable connections may play an important role, but obviously we cannot analyze these. The institutional setup and a number of tests such as showing the absence of pre-trends support the view that the region of work of a Director is plausibly exogenous to her nomination decision. However, we cannot rule out potential region-specific time-variant unobservables that are correlated both with the probability to lend and the nomination decision. One major candidate is regional demand for loans, which may in principle respond to the nomination decision.

This paper contributes to several strands of literature. First, it is related to work on the political economy of international organizations, which focuses on the Unitefd Nations (UN), the International Monetary Fund (IMF) and the World Bank (WB), among others. This literature typically finds that political economy factors are major determinants behind important decisions at these institutions (for a review, see, Dreher and Lang 2019). For example, a number of studies show that the probability of receiving IMF and WB loans (as well as the leniency of the attached conditions) is positively correlated with the recipient countries’ voting behavior at the international arena, such as whether they vote in line with the US or G7 countries at the UN Security Council (see, among others, Stone 2004; Barro and Lee 2005; Sturm and de Haan 2005; Dreher et al. 2009; Kilby 2009; Kaja and Werker 2010; Moser and Sturm 2011; Dreher and Sturm 2012). This literature on international organizations has paid surprisingly little attention to the EIB, which is surprising given the size of the Bank. Robinson (2009) and Clifton et al. (2018) and Mertens and Thiemann (2019) are the few papers on EIB that we are aware of. These papers describe the Bank, its functions, and evolution using a mix of qualitative and quantitative methods. Our contribution is to bridge this gap.

Second, the paper adds to the related literature studying the politics behind different financing instruments of the EU (for general reviews, see, Alesina et al. 2005; Baldwin and Wyplosz 2012; Dür et al. 2020). In particular, studies find that political factors, such as voting and proposal powers of the Member States in the EU, but also other international organizations, systematically affect the allocation of the EU Budget (see, among others, Bachtler and Mendez 2007; Aksoy 2010; Aksoy 2012; Bodenstein and Kemmerling 2012; Schneider 2013; Mikulaschek 2018; Gehring and Schneider^2018^).^8^ Our contribution to this literature is to document the existence of home-bias at the regional level in addition to the previously found biases at the national level. This is important since regional home-bias may have very different implications. In addition, the failure to account for regional home-bias of EU level politicians might have led the previous papers to wrongly attribute these type of bias to national bias since the home regions of politicians are often situated within their home countries.^9^

Third, the paper contributes to a recent strand of mostly development-related research on regional favoritism. The literature shows that political leaders systematically give favors to their ethno-linguistic groups (Kudamatsu 2007; Miquel 2007; Franck and Rainer 2012; Kramon and Posner 2016; 2013; Dickens 2018) and their regions of origin (Do et al. 2017; Dreher et al. 2019) in terms of higher federal transfers and public goods, or as observed in higher intensity night light data more generally (Hodler and Raschky 2014). Golden and Min (2013) presents an overview of this literature. Following several recent extensions of these results to democracies (see, e.g., Carozzi and Repetto 2016; Fiva and Halse 2016; Baskaran and Lopes da Fonseca 2018; Fabre and Sangnier 2017; Dahan and Yakir 2019), for evidence on Germany, Italy, Israel, France and Norway, respectively), we show that favoritism also takes place in institutionally mature environments.

Finally, this paper is related to a field in financial economics studying whether political considerations influence credit allocations of government-owned banks. This literature finds that, unlike private banks, lending by government-controlled banks is likely to follow political business cycles and to flow to electorally important districts both in advanced (e.g., Chavaz and Rose 2019 and Englmaier and Stowasser 2017 with evidence on US and Germany, respectively) and in less developed countries (among others, see Dinc 2005; Cole 2009; Carvalho 2014, for evidence on Brazil, India and a set of 36 countries, respectively).^10^

## Institutional setting and data

### The European Investment Bank

The EIB was founded in 1958 following the Treaty of Rome. One of the important aims of the Bank from the very start was to support the EU in reaching its goals of integration. The annual sum of signed loans has risen substantially over time from 34 million Euros in 1959 to around 77 billion Euros in 2015, this positive trend kicking off especially from the 1980s.^11^ The EIB mainly lends to EU Member States (90% of signed loans in 2017, EIB2017b), but also to other countries all over the world. Today, the bank is the largest multilateral lending (and borrowing) institution in the world. It is the main EU funding source for some policy areas like transport, and, for some countries, EIB funds are larger than resources flowing from EU regional policies (Robinson 2009). A significant portion of the funds goes to poorer regions. The EIB’s target is to lend to projects related to cohesion spending at the amount equivalent to 30% of its annual new operations in the European Union, Pre-Accession and EFTA countries (EIB 2020b).

Applicants for a loan can be from all levels of government, as well as private and public firms. Projects that cost less than 25 million Euros are disbursed via intermediate banks. As the Bank of the EU, the EIB generally finances projects that are in line with the economic policy objectives of the EU. Currently, some of the main priorities of the Bank include support to innovation activities, small and medium sized enterprises, infrastructure projects, and projects enhancing sustainable environment (EIB 2020a).^12^

**Fig. 2:**
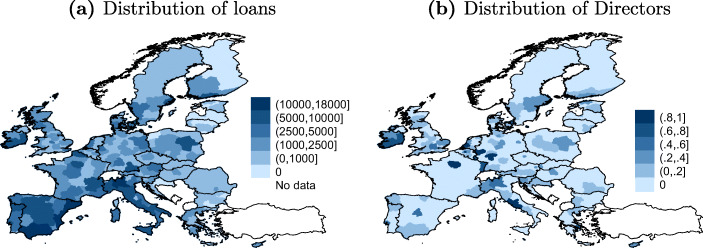
Distribution of EIB loans across European regions. Map **a** plots the total sum of EIB loans in million Euros that the respective region received within the period 1959-2015. Data source: EIB (2020c). Map **b** plots the share of years a region had (at least one) representative at the EIB Board of Directors within the period 1959-2015

Figures 1 and 2a present the size and targets of the EIB project loans. Fig. 1a shows that the loans have been growing substantially in size over time. Sub-figure (b) shows the distribution of loans across sectors. Infrastructure is the largest sector. When looking at the distribution of the total amount of loans over countries in Sub-figure (c), the major shareholders of the Bank seem to receive the largest shares of the EIB loans. Sub-figure (d) shows the average annual share of EIB loans over GDP from 1999 to 2014 for EU Member States. On average over this period, the largest recipient is Estonia, receiving funding amounting to more than 1% of its GDP, while the Netherlands gets the least with about 0,1% of GDP. Finally, Fig. 2a shows the geographical distribution of loans on the regional level. This map demonstrates substantial heterogeneity across regions with the distribution being skewed to the poorer Southern regions of the EU.

### Board of Directors and the approval of loans

Each EU Member State appoints one representative Director while an additional Director is nominated by the Commission. Moreover, today, there are 19 Alternate Directors. Most of the Directors are leading bureaucrats in their respective country, e.g., in the Ministry of Finance. Being a Director at the EIB is not a full-time job, they still follow their main occupation and only travel occasionally to the EIB in Luxembourg. The Directors are appointed for a period of five years and meet at least six times a year to decide on loan allocations. The Alternate Directors are also present at the meetings and support the Full Directors. The four big countries Germany, France, Italy, and the United Kingdom have two Alternates each, while all other countries share one Alternate in groups of two to eight.^13^

A loan is approved when at least one third of the Board members is in favor of the project and when these members represent at least 50% of the subscribed capital. The shareholders are the Member States. Each country’s share corresponds to the relative size of the country’s GDP in the EU at the time of joining the EU. Germany, France, Italy, and the United Kingdom each hold 16.1% of the total shares (EIB 2015a). With such significant weight, these four countries together can veto decisions.^14^

### Data on Directors’ careers

**Fig. 3:**
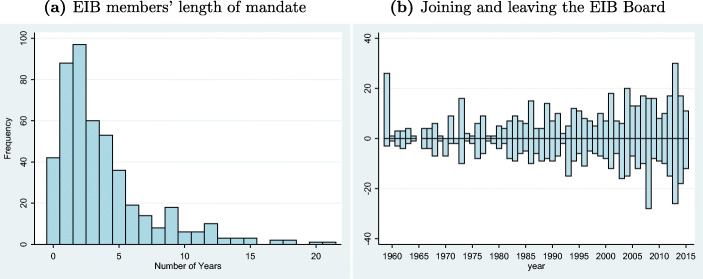
EIB members’ length of mandate over time. Figure **a** is a histogram on the number of years the Directors stay in office. Figure **b** plots the amount of Directors joining and leaving the Board per year, depicted on the positive and negative scale of the y-axis, respectively

The treated regions are defined based on a coding of the CVs of the EIB’s Board of Directors. Our sample includes 470 Board members from 1959 to 2015, including 254 full and 216 alternate members.^15^ Sub-figure (a) of Fig. 3 shows the length of the mandates of the Board members. The large bulk of Directors stayed two or three years in the Board. Sub-figure (b) represents a time line starting in 1959 showing the amount of Directors appointed to (positive values) and leaving (negative values) the Board per year. Finally, in Fig. 2b, we show the distribution of our treatment variable over space by plotting the share of years each region had one or more representative at the EIB Board. 84 regions are treated at least once. Comparing Fig. 2a and b, we can see a slight correlation of “darker” areas, i.e., between regions that have been treated more intensely and regions receiving more loans. Overall, the treated regions receive a share of 24% of the total project volume. 435 out of the 470 Directors come from regions including the capital city. 109 Directors work in lagging regions as defined by the regions receiving money from the European Cohesion Fund.^16^

**Fig. 4:**
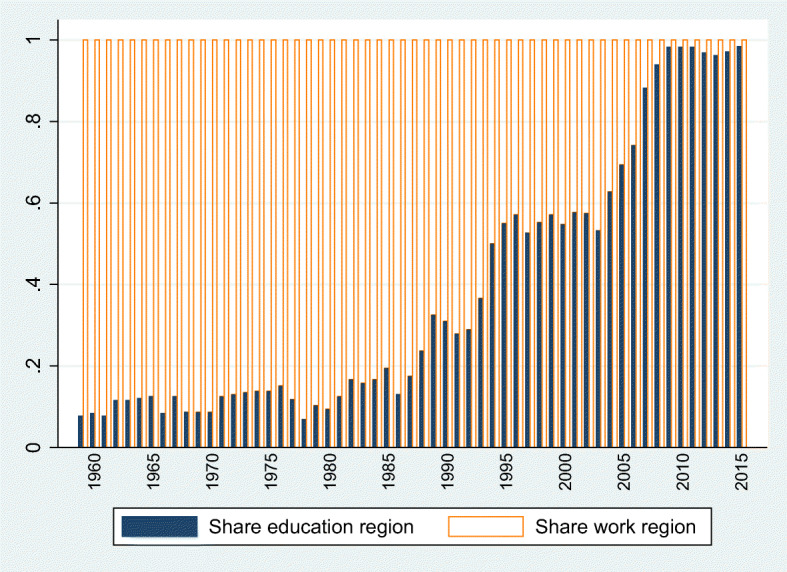
Availability of treatment variable. The bright and dark bars depict the share of Directors per year for whom we know the work and education region, respectively

We code the CVs of Board members in terms of the region they have worked when joining the EIB Board, and separately for the region where they obtained their highest degree of education. It would have been useful to also study the birth regions of Board members, however, such data is not available for privacy reasons. In the end, our preferred treatment variable is the work region dummy since we have complete information on this measure.^17^ For this variable, we have 36 regions that have been treated at some point in time. 19% of the overall loan volume are received by these 36 work regions. The education region, on the other hand, is missing for a substantial share of members that may introduce a downward bias in our estimates (since missing information is coded as 0, thus inflating the control group upwards, assuming that there is a home-bias in EIB lending). Figure 4 shows the share of Board members for whom we know the respective work and education regions. We use the education definition in Section 5 to study whether the timing when preferences towards home are realized matter for the interpretation of our results.

Throughout the paper, we exclude the Brussels region, as it is quite a special region in the European context. being the home of many European institutions.

### Data on EIB lending

The data on EIB loans goes back to 1959 and is publicly available on the website of the EIB (EIB 2020c). The website provides information on the size of the loan, the country, the sector, and the exact date when the contract was signed. Information on the region (either NUTS 1, NUTS 2 or NUTS 3) was provided to us by the EIB directly. This enables us to conduct a detailed analysis on a sub-national level.

Table 1 shows the availability of the loan data. Full information is available on the country level. Here, the total size of loans amounts to nearly 787 billion Euros. The number and total size of projects decrease the more we zoom into countries, i.e., as our focus becomes confined to smaller administrative units. One reason for less observations in the smaller administrative units is that some projects are allocated on higher administrative units and we do not know whether and how the money is distributed among sub-regions. The difference between number of projects and number of items comes from the fact that some loans within one project flow to several regions.

**Table 1:** EIB loans aggregated to different region definitions

Level	Sum in billion EUR	No. of items	No. of projects
Country	787.40	15,932	6,495
NUTS 1	578.71	14,010	5,366
NUTS 2	476.10	12,709	4,830
NUTS 3	285.25	7,917	3,443

### Other data

Regional data on control variables is taken from the European Regional Database (ERD) by Cambridge Econometrics. The dataset starts in 1980, however, Central and Eastern European countries^18^ as well as Malta and Cyprus are only available from 1990 onward. Our control variables are GDP, population size, hours worked, compensation of employees and gross fixed capital formation. Summary statistics of these and all other variables are collected in Table 2. For most countries we rely on the NUTS 2 region. For Estonia, Latvia, and Lithuania, we use the NUTS 3 region to get some sub-national variation, as their NUTS 2 regions correspond to the whole countries. For Cyprus and Luxembourg, even the NUTS 3 region corresponds to the entire country, thus leaving no variation for us to explore given our country-by-time fixed effects.

**Table 2:** Summary statistics of variables

Variable	Obs	Mean	Std.Dev.	Min	Max	Start	Source
EIB loan dummy	16,530	0.251	0.434	0	1	1959	EIB
EIB loans in million EUR	16,530	28.70	99.26	0	1874	1959	EIB
EIB loans over sum of EIB loans in year *t*	16,530	0.00345	0.0148	0	0.471	1959	EIB
EIB loans over GDP	9,251	0.00139	0.00397	0	0.0733	1980^a^	EIB
EIB loans interquartile range	4,148	2.550e + 07	5.860e + 07	0	9.850e + 08	1959	EIB
EIB loans standard deviation	2,418	3.220e + 07	4.890e + 07	0	6.970e + 08	1959	EIB
Ln loans without zeros	4,148	3.797	1.546	-2.717	7.536	1959	EIB
Capital city * joining EU	16,530	0.0457	0.209	0	1	1959	
GDP in billion EUR	9,251	35.57	45.27	0.271	565.0	1980^a^	ERD
GDP p.c. *t* − 6 to *t* − 1	8,960	18822	10118	1708	96309	1980^a^	ERD
Population in thousand	9,249	1694	1477	22.76	12070	1980^a^	ERD
Thousand hours worked per employee	8,686	1310	1150	19.43	9572	1980^a^	ERD
Compensation per employee in thousand EUR	8,726	18236	21533	219.6	246062	1980^a^	ERD
Gross fixed capital formation in million EUR	8,736	7763	9279	46.97	124611	1980^a^	ERD
Home region dummy work	16,530	0.0492	0.216	0	1	1959	EIB, EU Archives
Home region dummy education	16,530	0.0454	0.208	0	1	1959	EIB, EU Archives
Spatial lag Home region dummy work	16,473	0.0525	0.0298	0.0132	0.198		EIB, Eurostat
Ln sum EU funds	16,530	6.474	8.479	0	21.60	1994	EU Archives

^a^ For Eastern European countries, the variables are only available since 1990

Notes: ERD stands for European Regional Database

## Methodology

### Difference-in-differences

We estimate the following difference-in-differences (diff-in-diff) model:

1 $$  EIB\_loans_{ijt} =\alpha_{1} + \beta_{1} \cdot Home_{ijt} + \gamma_{1} \cdot \mathbf{X_{ijt}} + \psi_{ij} + \mu_{tj} + \epsilon_{ijt} $$

The index *i* stands for the region in country *j*, and year *t*. *EIB*_*loans*_*ijt*_, our outcome variable, is either a dummy to measure the extensive margin of receiving an EIB loan, or the natural logarithm of the amount of EIB loans. To combine extensive and intensive margin analyses, we use a Poisson pseudo-maximum likelihood (PPML) model with two different dependent variables: the loans-GDP-ratio and the share of loans a region received in total amount of loans in a given year.

*Home*_*ijt*_ is our main variable of interest. This variable measures whether a region had at least one representative at the EIB’s Board of Directors at a given point in time. Region-year observations are coded as treated (*Home*_*ijt*_) whenever a person currently part of the Board either studied or is currently working in the given region (as reported in the Board of Directors’ CVs). With 28 Full Directors representing one EU Member State each, one Full Director from the Commission, and 19 Alternate Directors, who are elected for a term of five years, we have a good degree of both cross-regional and cross-time variation in the treatment variable (see Fig. 2).

*X*_*ijt*_ is a vector of control variables.^19^ We also include region fixed effects (*ψ*_*ij*_) and country-by-year fixed effects (*μ*_*tj*_). These two-way fixed effects help us capture several potential endogeneity issues in the allocation of loans. Region fixed effects allow us to control for time-invariant region-specific factors. Importantly, our design with regional variation allows the inclusion of country-by-year fixed effects, which account for time-variant macroeconomic shocks such as national fiscal and monetary policy changes that affect countries differently but regions within a country similarly.

### Distributed lag model

We use a distributed lag model to study the timing of the effect of having a representative at the EIB Board on lending. In so doing, we include pre-trends of joining and lags of leaving the Board and, following Fabre and Sangnier (2017), separate treatment dummies for the first three years the Board member is in office and a fourth dummy for the remainder of the time in office. The equation is as follows:
2

The expression ${\sum }_{w=-4}^{-1} \beta _{w} 1st\_year\_Board^{w}_{ijt}$ defines the four pre-trends of the entry of each Board member. ${\sum }_{w=1}^{3} \gamma _{w} in\_office\_year^{w}_{ijt}$ are dummies for the first, second and third year in office. is a dummy for being in office the fourth and any further year. Finally, ${\sum }_{w=4}^{1} \delta _{w} L_{w} last\_year\_Board_{ijt}$ stands for four lags of the exit of the Board. The rest of the variables are the same as in Eq. 1.

## Results

### Baseline results

**Fig. 5:**
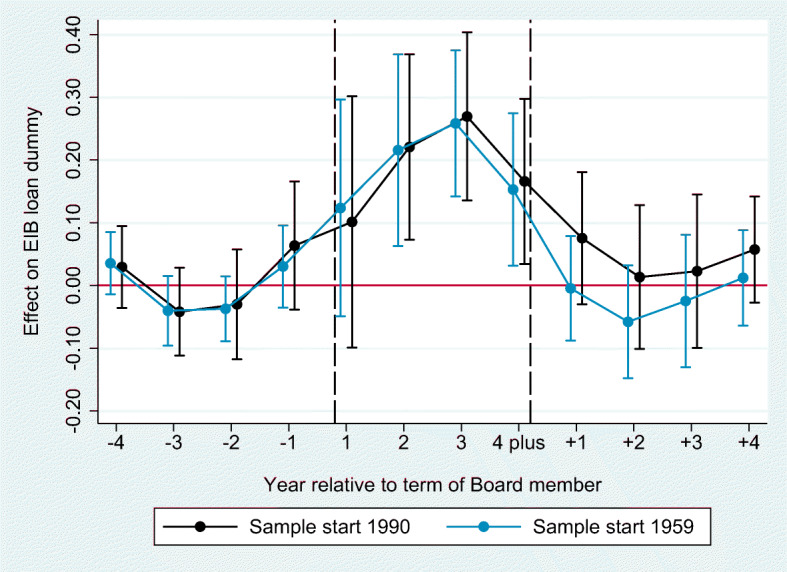
Probability of receiving a loan around the time of joining and leaving the EIB Board. This graph presents the estimation of Eq. 2. The corresponding regression table can be found in Table 7 columns 1 and 2

In this section we present our baseline analysis on whether regional favoritism affects the distribution of EIB lending to European regions. We start by discussing the estimation results of the difference-in-differences model as shown in Table 3, then proceed to discussing the results of the distributed lag model as plotted in Fig. 5. In both cases the treatment variable captures whether a region has a “representative” in the EIB’s decisive body, its Board of Directors. This variable takes a dummy equal to one if at least one Board member has worked in a given NUTS 2 region, and is 0 otherwise.

**Table 3:** Baseline: regional favoritism in the allocation of EIB loans

	(1)	(2)	(3)	(4)	(5)	(6)	(7)	(8)
VARIABLE	EIB Loan Dummy					Ln Loans	Loans / GDP	Loans / Tot. Loans
Work region dummy	0.1457***	0.1768***	0.1712***	0.2064***	0.1776***	-0.0256	0.9834***	0.6050**
	(0.0487)	(0.0462)	(0.0521)	(0.0762)	(0.0513)	(0.2533)	(0.3124)	(0.2786)
Ln population			0.4608*	-0.3558	0.6064**	1.4334	-0.2290	-0.5164
			(0.2381)	(0.6956)	(0.2351)	(1.2371)	(1.0254)	(0.8516)
Ln GDP			0.1299	-0.4264***		2.1337***		2.7845***
			(0.1337)	(0.1635)		(0.7535)		(0.5775)
Ln GDP p.c. *t* − 6 to *t* − 1					0.1204			
					(0.1228)			
Hours worked per employee			0.4395*	-0.2512	0.4527*	0.1459	-0.0700	-2.1312*
			(0.2427)	(0.3430)	(0.2437)	(1.8445)	(1.0652)	(1.1477)
Compensation per employee			0.0009	0.0061	0.0016	0.0205	-0.0523***	-0.0305*
			(0.0045)	(0.0048)	(0.0045)	(0.0171)	(0.0192)	(0.0168)
Ln gross fixed capital formation			-0.0794	0.1253	-0.0755	0.1262	1.4807***	0.7588***
			(0.0511)	(0.0822)	(0.0532)	(0.3782)	(0.2297)	(0.2746)
Observations	16,530	7,540	6,642	6,642	6,581	2,782	6,442	6,442
R-squared	0.3844	0.2557	0.2540	0.3268	0.2526	0.2992	0.1910	0.4429
Number of regions	290	290	266	266	266	258	258	258
Mean of dependent variable	.25	.39	.42	.42	.42	4.18	.0017	.0039

*** p < 0.01, ** p < 0.05, * p < 0.1. The table presents results for the estimation of the model in equation 1. Standard errors are in parentheses and clustered on the level of NUTS 2 regions. The sample start year is 1990 for all regressions except column 1, where it is 1959. The method in all columns is OLS except for columns 7 and 8, which use PPML. Region fixed effects are included in all regressions. Country-year fixed effects are in included in all columns except 7 and 8, where only year fixed effects are included. Column 4 includes a region-specific time trend. Given the dependent variables, columns 1 to 5 analyze the extensive margin, column 6 the intensive margin, and columns 7 and 8 combine the intensive and extensive margin

Columns 1-5 of Table 3 show the extensive margin results for the work region, where the dependent variable is a dummy indicating whether a region received at least one EIB loan in a given year or not. The evidence supports the hypothesis that the treated regions receive more EIB loans compared to regions that do not have a representative at the EIB Board. This extensive margin effect is robust across the specifications 1 to 5 of Table 3.^20^ The size of the effect is an increase of 14 to 21 percentage points in the likelihood of receiving a loan. The average underlying probability that a region receives any lending is between 23% and 42%. Therefore, the home-bias effect amounts to a large 40-61% increase in the probability of lending compared to the sample mean.

In column 6 of Table 3, we study the intensive margin, that is we ask whether treated regions receive larger EIB loans given that they received at least one loan. For the dependent variable, we take the (log) size of all lending aggregated to the region-year level. As a result regions with no EIB loans in a certain year are dropped from the sample. The estimated intensive margin result is small and not distinguishable from zero.

Columns 7 and 8 of Table 3 combine the extensive and intensive margins and use a Poisson pseudo-maximum likelihood (PPML) estimator.^21^ As outcomes variables, we use the loans-over-GDP ratio as well as study the share of loans in total loans. The home-bias effect is positive and statistically significant in both cases. The sizes of the coefficients are interpreted as a 98% increase in the loans-over-GDP ratio and a 61% percent increase in the share of loans in total loans, respectively. This effect is larger than the one for the extensive margin, however, the sample means in these specifications are very small, 0.17% and 0.39%, respectively.

We now proceed to the estimation results of the distributed lag model as specified in Eq. 2. Given the findings of Table 3 we focus on the extensive margin response. In particular, we are interested in the timing of this effect. Figure 5 shows that the treatment effect becomes positive and remains so during the whole tenure. The point estimates are large^22^ and statistically significant in all but one year of tenure. Once the Director has left the Board, the point estimates drop almost immediately and are not statistically significant anymore indicating that the home bias effect is only present during the Director’s tenure. The immediate effects is plausible because our outcome variable measures lending commitments rather than their actual disbursement. The trends prior to the treatment are not significantly different from zero in any of the four lags that we estimate.^23^ The full regression results corresponding to Fig. 5 are reported in Table 7.

Figure 5 replicates the estimation for both the full sample since 1959 without control variables and the post-1990 sample where the control variables are available. The results look very similar, which is not surprising given that the EIB started to lend actively starting from the 1980s. Therefore, we rely on the post-1990 sample and benefit from the availability of control variables for all of our analyses that follows.

### Robustness tests

#### NUTS definition and clustering

To check the robustness of our results, we redo the analysis for the extensive margin using the treatment dummy by clustering on the NUTS 1 region instead of the NUTS 2 region, and by defining the regions as NUTS 3 or NUTS 1 regions. The results are collected in columns 1-3 of Table 4, and are robust to our baseline results.

**Table 4:** Robustness checks

	(1)	(2)	(3)	(4)	(5)	(6)	(7)	(8)	(9)	(10)
VARIABLES	EIB Loan Dummy									
Work region dummy	0.1712***	0.1891***	0.1758***	0.0896***	0.1705**	0.1871**	0.4960***	0.1747***	0.1836***	0.3672***
	(0.0546)	(0.0527)	(0.0441)	(0.0272)	(0.0789)	(0.0909)	(0.1362)	(0.0518)	(0.0546)	(0.0840)
Capital city * joining EU					0.0011					
					(0.1021)					
Ln ESI funds								0.0057		
								(0.0041)		
Spatial lag									0.6903	
									(0.7894)	
Sample definition	full	full	full	full	full	no capitals	only capitals	full	full	full
Observations	6,642	34,164	2,269	29,848	6,642	6,018	624	6,642	6,642	27,872
R-squared	0.2540	0.1007	0.4502	0.4050	0.2540	0.2584	0.9119	0.2542	0.2541	
Number of regions	266	1,314	91	287	266	241	25	266	266	269
Mean of dependent variable	.42	.1	.67	.17	.42	.41	.48	.42	.42	.18

*** p < 0.01, ** p < 0.05, * p < 0.1. The table presents results for the estimation of the model in Eq. 1. Standard errors are in parentheses and they clustered on the level as indicated in the table. The sample start year is 1990 for all regressions. The method is OLS for all regressions except column 10, where it is Logit. Region fixed effects are included in all regressions. Control variables are included in all regressions except columns 2, 4, and 10. Country-year fixed effects are included in all regressions except 4 and 10. Year-quarter fixed effects are included in column 10. Country-year-quarter fixed effects are included in column 4

#### Quarterly analysis

To further test the robustness of our results, we disaggregate the data to the quarterly level. This data enables us to include country-by-year-by-quarter fixed effects that further increase the validity of our results by controlling for the business-cycle on the national level. Column 4 of Table 4 shows the results for the extensive margin. The effect is smaller in size but still of substantial magnitude and statistically different from zero.

#### Joining the EU

Another concern with our analysis has to do with the fact that the EU has expanded in several waves within our sample period. Even though we control for region and country-year interacted fixed effects, this expansion may still be an issue if the treatment regions of the small countries that join the EU are concentrated in the capital and if these regions simultaneously benefit disproportionally more from joining the EU. Thus, in Table 4 column 5, we include an interaction term between capital cities and a post-EU dummy. The result is nearly identical with the baseline result in Table 3 column 3.

#### Capital cities

As can be seen from Fig. 2, the capital cities are more likely to be treated than other parts of countries while being economic centres they are also more likely to be receiving loans from the EIB. Although this potential confounding effect should be fully accounted for by the region fixed effects, we do two additional robustness tests by excluding all regions with capital cities from the sample, or by only including these regions in the sample. The results are collected in Table 4 columns 6 and 7. In both instances we observe a positive and significant treatment effect.

#### EU structural and investment funds

A further concern relates to the fact that a large share of EIB loans co-finance the EU Structural and Investment Funds (ESI Funds). While we controlled for the potential eligibility for such funds in Table 3 column 5, we now conduct a more direct test. We want to analyze whether our baseline results still hold when we control for the fact that regions received some of the ESI Funds. We rely on annual disbursement data of the following four ESI Funds: the European Regional Development Fund, the Cohesion Fund, the European Social Fund, and the European Agricultural Fund for Rural Development. We opt for a simultaneous disbursement of ESI Funds and EIB treatment effects, well aware that lagged specifications might be appropriate as well. The results in column 8 of Table 4 show no distortion of the main treatment effects.

#### Spillover effects

Furthermore, we test whether neighbor regions of treated regions also have an increased probability of receiving loans. For that purpose, we create a spatial lag where we weight the home region dummy of the other regions by their inverse distance. The results collected in Table 4 column 9 do not show evidence of regional spillover effects.

#### Excluding countries and time periods

We analyze whether specific countries or time periods drive the baseline results. Table 8 shows the baseline estimates by dropping the 28 EU Member States one-by-one. The estimated treatment effect is fairly stable in size (the point estimate varies from to 0.1543 to 0.1926) and is always significantly different from zero at the 1% level. Thus, it seems that no specific Member State is solely responsible for the baseline result.

Similarly, Table 9 drops decades or five-year periods one-by-one. When the period from 1999 to 2008 is excluded, the treatment effect reduces by about twice in size and becomes statistically indistinguishable from zero. However, when we exclude the first and second five years of this decade separately, both effects are positive and significantly different from zero. Therefore, we conclude that although it seems that much of the favoritism that we document may be coming from the post-1999 period, we cannot say that the result is solely driven by this period.

#### Model choice

We have so far used simple linear probability models when studying the extensive margin response, and Poisson pseudo-maximum likelihood models in specifications that combine the extensive and intensive margin responses (see Table 3). Since our dependent variable in the extensive margin specifications is a dummy variable, we can interpret the expected value of the estimate as a probability. Non-linear models provide potential benefits when the underlying model is highly non-linear. At the same time they lead to considerable complications including the incidental parameter problem as well as computational difficulties. These problems especially aggravate in our case, which models a large set of fixed effects, in particular due to the inclusion of country-by-year fixed effects. Moreover, the main disadvantages of using a linear model, that are conceptual arguments against linearity or the argument that predictions might lie outside of the theoretically possible range of (0,1), do not materialize in our case. We, therefore, estimate one computationally plausible non-linear model as a robustness test, but keep the linear models as our baseline estimator. In particular, in Table 4 column 10, we specify a logit regression without country-by-time fixed effects on quarterly level data. The quarterly data has a longer time-series than the annual data which somewhat downplays the incidental parameter problem. The marginal effect of the treatment effect is slightly larger than that of the baseline model but consistent in direction and significance.

#### Few treated clusters

**Fig. 6:**
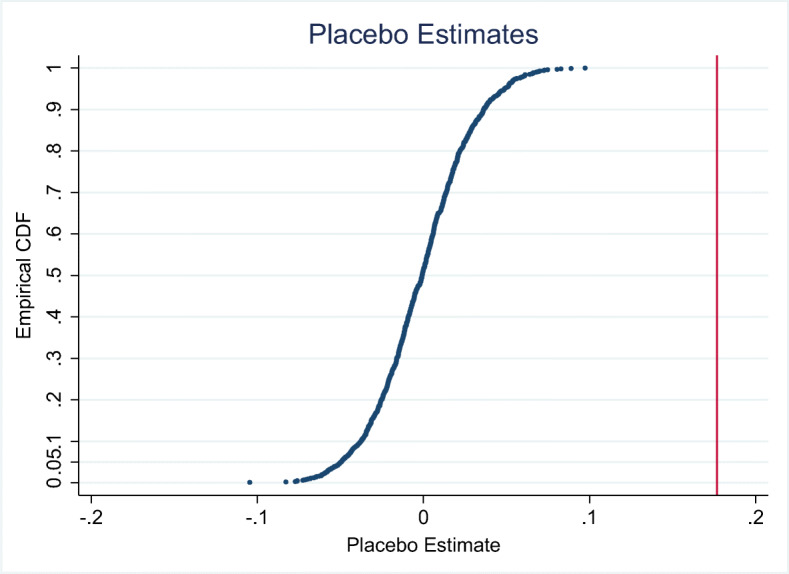
Randomization inference. The graph plots the cumulative distribution function on the y-axis and the placebo treatment effects on the x-axis. The vertical line indicates our treatment effect reported in Table 3 column 3

One problem with our setting could be that the number of treated clusters is small. With few treated clusters, inference problems can arise because the large-sample approximations for inference are no longer applicable (Conley and Taber 2011). As the share of treated region-years is 7% for the post-1990 sample, we conduct randomization inference by randomizing the treatment. In the original dataset, the treated observations are distributed across 36 of 291 regions. Therefore, we conduct a two-step randomization of treatment where in the first step 36 regions are randomly assigned to be treated regions. Among them, we again choose the same number as treated observations as in the “real” sample before running the specification. This randomization inference mechanism is conducted for 1000 replications, which gives us 1000 placebo treatment effects. We compute the cumulative distribution function of these placebo effects and compare it to our treatment effect. The resulting graph is depicted in Fig. 6. The graph shows that our result is rare and that the few treated clusters are unlikely to give rise to issues in the sense of Conley and Taber (2011) in our analysis.

### Result heterogeneity

#### Governing bodies

**Fig. 7:**
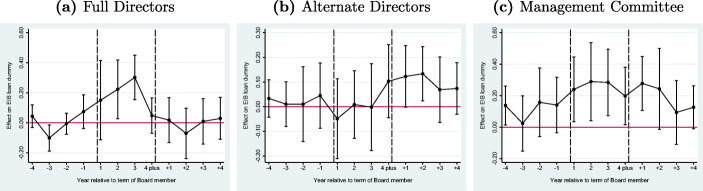
Full and alternate directors and management committee. These graphs present three estimations of Eq. 2 where the the treatment effect is the work region of: **a** Full Directors, **b** Alternate Directors, and c) Members of the Management Committee. The corresponding regressions can be found in Table 7 columns 3-5

The EIB Board has 29 Full and 19 Alternate Directors. As the name suggests, Alternate Directors mostly assist the work of the Full Directors. Also, Full Directors represent an individual Member State while most Alternate Directors represent a group of countries. It is, therefore, our expectation that the home-bias of Alternate Directors is smaller than that of the Full Directors. In Fig. 7a and b, we replicate the analysis for Full and Alternate Board members separately. As expected, we see large and statistically significant point estimates of the treatment effect for the Full Directors but not for the Alternate Directors. The positive lags for the Alternate Directors after exiting EIB can be explained by the fact that some Alternates become a Full member after their term as Alternate.

The Management Committee is the executive body of the EIB, and currently consists of one President and eight Vice-Presidents. Since the foundation of the EIB, there have been 54 members of this Committee in total including seven Presidents. We have data on the work region prior to joining the EIB of 49 Committee members. Even though the Management Committee of the EIB has no direct influence on the approval of loans, anecdotal evidence suggests that these are generally influential positions within the EIB. For example, Counter Balance (2016) reports, for instance, that Philippe de Fontaine Vive, a Vice-President at the EIB from 2003 to 2015, joined CMA-CGM, a container shipping conglomerate, which received a public-private partnership contract from EIB. He is now on the board of BMCE Bank, which has a long association with EIB. Counter Balance (2016) also reports about Gillian Day who held senior positions at the EIB at the same time when EIB has awarded numerous loans to the Royal Bank of Scotland where she served as Managing Director until February 2015. Moreover, this body together with the staff is also responsible for preparing the documents for the loan approval by the Board of Directors. Figure 7c plots the analysis for the Management Committee. We observe positive and statistically significant effects in the treatment period, which would lend support to the hypothesis that the Management Committee not only has influence on loan approvals but also engages in home-bias lending. However, we also note that the confidence intervals in this exercise are quite large owing to the small number of Management Committee members.

## Potential mechanisms

After having documented the body of evidence on the existence of the home-bias effect in lending, the crucial question is whether these discriminatory lending practices facilitate economic efficiency or whether they result in inefficient misallocation of resources. As discussed in Section 1, the two main mechanism that predict a larger flow of transactions into the home regions are that this lending is either driven by favoritism or by the information advantage of Directors. Both of these potential mechanisms are consistent with our evidence, however, they have divergent predictions on the economic value of these transactions. While favoritism-driven lending will arguably lead to resource misallocation, lending due to a potential information advantage of Directors regarding their home regions may enhance efficiency. The net welfare effects of home-bias can therefore be both positive or negative depending on the relative strength of either of these two channels. In this section, we design a number of indirect tests to try to isolate the two motives behind lending decisions.

### Sector and project size

First, we study potential treatment heterogeneity along the size distribution of loans as well as along six broad sectors that our data captures.^24^ This exercise allows us to identify on a more granular level the types of projects that drive the finding of home-bias. This is an interesting exercise by itself, and may additionally be informative about the mechanisms in play.

For the latter case of sectors, we estimate Equation 1 with the dependent variable being a dummy of having at least one loan in a certain sector. The results are collected in Table 5. Column 1 shows the treatment effects by sector for the extensive margin independent of the project size. The home-bias is only present for infrastructure projects. The effect of 13 percentage points translates to an increase of 46% compared to the sample mean, which is in line with our baseline result. We then we split the approved loans into quartiles according to their size within each sector. To control for differences between poor and rich countries, and size effects over time, we classify the loans into quartiles depending on the country and decade. Columns 2 to 5 of Table 5 show the treatment effects per quartile and sector. For the infrastructure sector, the treatment effects are positive and statistically significant for projects above the median.^25^

**Table 5:** Heterogeneous effects according to sectors and loan sizes

			(1)	(2)	(3)	(4)	(5)	(6)		(7)	
	no.^a^	mean^a^	Loan dummy						Obs		Obs.
VARIABLES			all	1st q.tile	2nd q.tile	3rd q.tile	4th q.tile	no. loans		ln loans	
Total	8,772	48.21	0.1712***	0.0179	0.0602*	0.0575	0.1274**	0.7976***	6,442	-0.0256	2,782
			(0.0521)	(0.0421)	(0.0331)	(0.0558)	(0.0625)	(0.2688)		(0.2533)	
Agriculture	28	35.62	0.0019	0.0010	0.0011	0.0012	0.0023	32.5068*	108	omitted	17
			(0.0020)	(0.0009)	(0.0011)	(0.0011)	(0.0020)	(16.7606)			
Energy	1,812	39.71	-0.0246	-0.0064	-0.0286**	-0.0204	-0.0283	0.6201***	4,667	-0.4318	895
			(0.0346)	(0.0138)	(0.0138)	(0.0222)	(0.0295)	(0.2064)		(0.5369)	
Industry	1,609	49.48	0.0091	-0.0275	-0.0429*	-0.0299	0.0431	-0.1088	4,917	0.2310	908
			(0.0431)	(0.0279)	(0.0242)	(0.0251)	(0.0318)	(0.2687)		(0.5254)	
Infrastructure	4558	49.61	0.1332**	-0.0017	0.0276	0.0814**	0.1528***	1.2456***	5,968	0.7775***	1,859
			(0.0522)	(0.0288)	(0.0303)	(0.0397)	(0.0539)	(0.3308)		(0.2884)	
Non-Market Services	472	70.87	0.0729	0.0027	0.0165	0.0529*	0.0018	0.4562	2,598	-2.2379	341
			(0.0465)	(0.0052)	(0.0125)	(0.0285)	(0.0221)	(0.4374)		(0.6550)	
Services	293	36.65	0.0046	-0.0104*	-0.0007	0.0102	0.0157	0.1158	2,494	-0.0313	217
			(0.0329)	(0.0060)	(0.0186)	(0.0095)	(0.0162)	(0.5886)		(0.7934)	
Observations			6642	6,642	6,642	6,642	6,642	-		-	
Number of regions			266	266	266	266	266	-		-	

^a^ No. corresponds to the number of projects per sector and mean to the mean project size in million Euros after 1990. *** p < 0.01, ** p < 0.05, * p < 0.1. The table presents results for the estimation of the model in Eq. 1. Standard errors are in parentheses and clustered on the level of NUTS 2 regions. The sample start year is 1990 for all regressions. Control variables, region fixed effects, and country-year fixed effects are included in all regressions. The method is OLS for all regressions except column 6, where it is PPML

Similar to our baseline analysis, columns 6 and 7 of Table 5 extend the sector-specific results to the intensive margin analysis of looking at, respectively, the number of loans and log loan sizes. Infrastructure is the only sector where we find significant treatment effects in both columns.^26^ The magnitudes are large and can be interpreted as an increase of 120% in the number of loans (or 0.9 more loans compared to the mean of 0.75 loans) or a 116% increase in the size of infrastructure loans. This evidence that on the intensive margin

Although not a direct test, this evidence that the home-bias phenomenon is driven by infrastructural mega-projects may hint towards favoritism rather than an informational channel, as the need for these large projects is rather common knowledge than the need for smaller and more sophisticated projects. Papers by Do et al. (2017) and Persson and Zhuravskaya (2016) find similar evidence for construction infrastructure in Vietnam and China, respectively.

### Job switchers

As a second test of mechanism, we collect additional data on the work regions of Directors after their service at the EIB,^27^ and limit the analysis to a sub-sample of Directors who switch jobs. We then separately study the probability of switchers to lend money to either their pre-EIB regions or the new post-EIB regions while in office at the Board. The assumption behind this test is that a Director who has not yet worked in a certain region does not have an information advantage about that region, while sending money to a pre-EIB region can be a mix of both the favoritism and information channels.

**Fig. 8:**
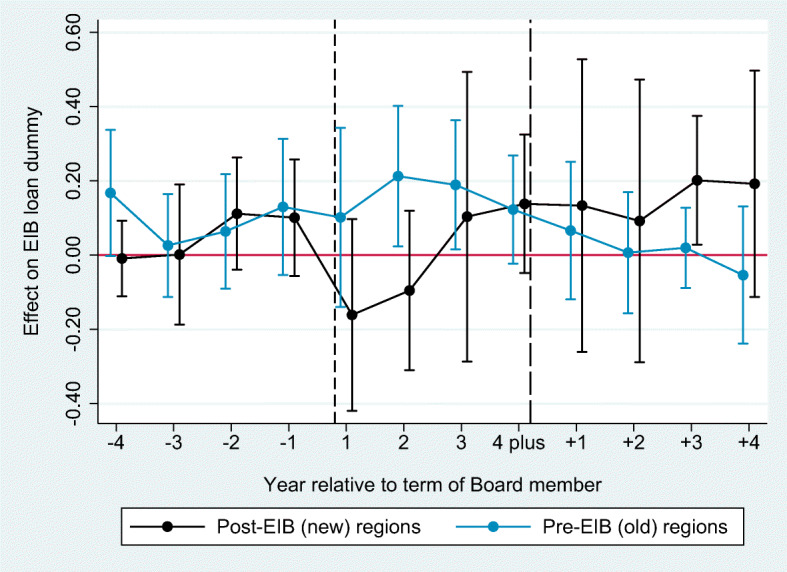
Tests for mechanisms: Job switchers. This graph presents the estimation of Eq. 2. The corresponding regression can be found in Table 7 columns 6 and 7

Figure 8 is estimated with Eq. 2 and identifies the timing of effects of job-switchers serving at the EIB Board on lending to either their pre-EIB (“old”) regions or their post-EIB (“new”) regions. The results for the old regions are positive and statistically significant in the treatment, but not in the pre-treatment periods, which confirms our baseline result on this sub-sample limited to job switchers. If information advantage was the main mechanism at play, we would expect the entire home-bias effects to be driven by the transfers to old regions and nearly zero transfers flowing to the new regions. In contrast, Fig. 8 does not find a precise zero effect on transfers to these regions. The point estimates of lending to new post-EIB regions are increasing with tenure at the Board and they reach near-statistical significance at end of the period. This evidence is weak possibly due to the few number of switchers in our sample. However, at the very least, the evidence does not reject the null hypothesis that Directors are unable to send transfers to regions over which they are unlikely to have any observable informational advantages.

### Degree of information

Third, we follow (Rajan 1992; Persson and Zhuravskaya 2016; Fisman et al. 2017) and hypothesize that the degree of information a Director has about a region should be positively correlated with the amount of home-bias lending. Similar to this work, we measure the degree of information by Directors’ length of experience in the region measured in years.^28^ Column 1 of Table 6 adds a variable capturing the number of years in work regions to our baseline specification. Next, we limit the sample to treated observation only, and in columns 2 and 3 of Table 6 test whether treatment effects are heterogeneous in this sub-sample according to, respectively, the number of years of experience as a continuous variable or an indicator function specifying several intervals of the experience variable. None of these tests confirms the hypothesis that more experience, a likely correlate of information, drives more lending.

**Table 6:** Tests for mechanisms

	(1)	(2)	(3)	(4)	(5)	(6)	(7)	(8)	(9)
VARIABLES	Loan dummy			Ln IQR	Ln Std. Dev.	Loan dummy			
Work region dummy in t	0.1419**			0.4911	0.4392		0.1564***	0.1090**	0.1251**
	(0.0585)			(0.4908)	(0.3920)		(0.0585)	(0.0545)	(0.0496)
Education region dummy in t						0.0534	0.0340	0.0283	
						(0.0364)	(0.0390)	(0.0392)	
Experience in work region	0.0069	0.0061							
	(0.0054)	(0.0045)							
region experience - reference: 0 years									
1-3 years			-0.1150						
			(0.2361)						
4-6 years			-0.5602						
			(0.6629)						
7-9 years			0.0160						
			(0.2227)						
10-12 years			0.2301						
			(0.4267)						
12+ years			0.1995						
			(0.1242)						
Sample	full	treatment = 1		without zero loans		full	full	both regions known	
Observations	6,642	470	470	1,600	1,600	6,642	6,642	6,642	6,642
R-squared	0.2547	0.9042	0.9081	0.4003	0.3934	0.2528	0.2542	0.2537	0.2535
Number of regions	266	29	29	221	221	266	266	266	266
Dependent variable mean	.42	.68	.68	17.03	16.79	.42	.42	.42	.42

*** p < 0.01, ** p < 0.05, * p < 0.1. The table presents results for the estimation of the model in Eq. 1. Standard errors are in parentheses and clustered on the level of NUTS 2 regions. The sample start year is 1990 and the method is OLS for all regressions. Control variables, region fixed effects, and country-year fixed effects are included in all regressions

### Loan size dispersion

Fourth, we follow Cornell and Welch (1996) and Fisman et al. (2017) and hypothesize that informed Directors should have higher precision signals about the creditworthiness of borrowers originating from their home regions which would increase the variance of the distribution of priors across these borrowers. This hypothesis of higher variance of priors leads to the testable prediction that the dispersion of loan sizes flowing to home regions are higher than those of loans going to other regions. We adopt our baseline specification and instead of the dependent variable in columns 4 and 5 of Table 6 take as measures of loan dispersion the (log of) inter-quartile range and the standard deviation of loan sizes per region and year, respectively. The sample is limited to region-year observations having two and more loans. This evidence again fails to find any support that the information mechanism is the main driver of the home-bias lending.

### Social preferences

Ruling out the information channel as the main mechanism at play tells us that this type of lending is not likely to be welfare enhancing. However, even if we could entirely rule out the information mechanism, the favoritism explanation would not be the only remaining explanation. One competing explanation, as advanced for example by Do et al. (2017) in a different context, is that Directors may simply have social preferences towards their home regions. There may be various alternative explanations as well, but to the degree that we are aware of the literature this and other mechanism are all likely to lead to misallocation of resources.

Nevertheless, in a final step, we follow (Persson and Zhuravskaya 2016) and exploit the timing of formation of the home-bias assuming that social preferences towards a region are likely to take shape before the last region of work and perhaps much closer to years of early adulthood. For a sub-sample of 263 Directors, we are able to code the regions of education. Columns 6 to 9 of Table 6 then asks whether the additional lending is flowing to regions of Directors’ workplace rather than their education regions.^29^ Our evidence seems to be driven by regions of workplace rather that of education, which may speak against the hypothesis that favoritism is driven by social preferences rather than personal gain.

## Conclusion

In this paper, we study political economy aspects of lending decisions within the European Investment Bank. This is an important extension of the literature on the political economy of international organizations for at least two reasons. First, the EIB is the largest multilateral lending institution in the world, and thus an important case study by itself. Second, the EIB is closely nested within the larger framework of the European Union institutions, thus this paper may have wider relevance for policy reform in the EU.

We document that Member State-nominated technocrats governing the Bank favor their home regions by allocating more EIB lending towards these regions. Our evidence does not provide a definitive answer to the crucial question of whether this bias is economically inefficient. However, we think that the question of whether favoritism plays a role in resource allocation decisions at the EU and particularly at the EIB deserves a further debate.

In particular, the EIB may benefit by increasing the level of transparency in its decision making processes. This is in line with ongoing calls to reform EIB institutions, such as by introducing stronger rules for the disclosure of conflict of interest by the EIB Board of Directors and other senior staff. This evidence also stresses the important role to be given to debates on institutional reforms for the EU’s various financing instruments before any further and more complex arrangements are established. The well-known accountability problems in the EU cannot be solved by simply delegating authority to technocrats who are often perceived to be rather independent of political constraints given the absence of electoral incentives.

## Electronic supplementary material

Below is the link to the electronic supplementary material.

(ZIP 10.4 MB)

(PDF 166 KB)

## Appendix

**Table 7:** Results from the distributed lag model

	(1)	(2)	(3)	(4)	(5)	(6)	(7)
VARIABLES	EIB Loan Dummy						
First year in office in *t* − 4	0.0353	0.0490	0.0441	0.0336	0.1377**	0.1674*	-0.0094
	(0.0251)	(0.0346)	(0.0382)	(0.0386)	(0.0626)	(0.0863)	(0.0516)
First year in office in *t* − 3	-0.0404	-0.0570	-0.1006**	0.0105	0.0236	0.0258	0.0012
	(0.0282)	(0.0376)	(0.0442)	(0.0462)	(0.0891)	(0.0704)	(0.0961)
First year in office in *t* − 2	-0.0373	-0.0203	-0.0053	0.0103	0.1575	0.0636	0.1113
	(0.0262)	(0.0481)	(0.0357)	(0.0771)	(0.1105)	(0.0783)	(0.0768)
First year in office in *t* − 1	0.0302	0.0638	0.0744	0.0450	0.1406	0.1297	0.1008
	(0.0334)	(0.0533)	(0.0567)	(0.0674)	(0.0897)	(0.0933)	(0.0798)
First year in office	0.1234	0.1266	0,1506	-0.0487	0.2400**	0.1015	-0.1615
	(0.0879)	(0.1100)	(0.1341)	(0.0822)	(0.1046)	(0.1227)	(0.1313)
Second year in office	0.2157**	0.1979**	0.2231**	0.0088	0.2894**	0.2126**	-0.0957
	(0.0778)	(0.0834)	(0.0990)	(0.0694)	(0.1261)	(0.0964)	(0.1090)
Third year in office	0.2584***	0.2717***	0.3025***	-0.0020	0.2845***	0.1890**	0.1034
	(0.0591)	(0.0761)	(0.0749)	(0.0895)	(0.1072)	(0.0884)	(0.1982)
Fourth year or more in office	0.1529**	0.1355*	0.0482	0.1033	0.1977**	0.1227*	0.1378
	(0.0619)	(0.0737)	(0.0595)	(0.0752)	(0.0929)	(0.0741)	(0.0948)
Last year in office in *t* + 1	-0.0047	0.0683	0.0182	0.1229*	0.2777***	0.0659	0.1333
	(0.0423)	(0.0540)	(0.0758)	(0.0632)	(0.0873)	(0.0941)	(0.2002)
Last year in office in *t* + 2	-0.0581	0.0210	-0.0701	0.1336**	0.2436*	0.0065	0.0918
	(0.0458)	(0.0581)	(0.0854)	(0.0560)	(0.1306)	(0.0830)	(0.1934)
Last year in office in *t* + 3	-0.0249	0.0166	0.0095	0.0691	0.0937	0.0194	0.2014**
	(0.0538)	(0.0637)	(0.0767)	(0.0676)	(0.1029)	(0.0550)	(0.0881)
Last year in office in *t* + 4	0.0120	0.0624	0.0292	0.0737	0.1261*	-0.0543	0.1919
	(0.0387)	(0.0431)	(0.0708)	(0.0529)	(0.0693)	(0.0939)	(0.1549)
Sample	Full and Alternate		Full	Alternate	MC	old regions	post regions
Observations	14,210	5,844	5,844	5,844	5,844	4,788	4,788
R-squared	0.3632	0.2563	0.2562	0.2535	0.2559	0.2200	0.2197
Number of regions	290	266	266	266	266	266	266
Mean of the dependent variable	.25	.41	.41	.41	.41	.42	.42

*** p < 0.01, ** p < 0.05, * p < 0.1. The table presents results for the estimation of the model in Eq. 2. Standard errors are in parentheses and clustered on the level of NUTS 2 regions. The sample start year is 1990 for all regressions except column 1, where it is 1959. Control variables, region fixed effects, and country-year fixed effects are included in all regressions

**Table 8:** Drop countries one-by-one

	(1)	(2)	(3)	(4)	(5)	(6)	(7)	(8)	(9)	(10)	(11)	(12)	(13)	(14)
VARIABLES	EIB Loan Dummy													
Work region	0.1641***	0.1708***	0.1518***	0.1712***	0.1909***	0.1543***	0.1709***	0.1712***	0.1845***	0.1746***	0.1679***	0.1672***	0.1712***	0.1669***
dummy	(0.0545)	(0.0521)	(0.0524)	(0.0520)	(0.0517)	(0.0563)	(0.0520)	(0.0521)	(0.0490)	(0.0528)	(0.0537)	(0.0520)	(0.0521)	(0.0546)
Without country	AT	BE	BG	CY	CZ	DE	DK	EE	EL	ES	FI	FR	HR	HU
Observations	6,417	6,392	6,492	6,617	6,442	5,700	6,517	6,642	6,317	6,167	6,517	5,992	6,642	6,467
R-squared	0.2501	0.2505	0.2525	0.2509	0.2445	0.2815	0.2483	0.2540	0.2615	0.2577	0.2467	0.2769	0.2540	0.2505
Number of regions	257	256	260	265	258	228	261	266	253	247	261	240	266	259
Mean of dependent variable	.42	.42	.43	.42	.42	.43	.42	.42	.42	.39	.42	.41	.42	.43
Work region	0.1712***	0.1806***	0.1712***	0.1712***	0.1712***	0.1712***	0.1710***	0.1596***	0.1713***	0.1658***	0.1606***	0.1898***	0.1926***	0.1629***
dummy	(0.0521)	(0.0532)	(0.0521)	(0.0520)	(0.0521)	(0.0520)	(0.0570)	(0.0545)	(0.0543)	(0.0551)	(0.0541)	(0.0519)	(0.0524)	(0.0558)
Without country	IE	IT	LT	LU	LV	MT	NL	PL	PT	RO	SE	SI	SK	UK
Observations	6,592	6,117	6,642	6,617	6,642	6,617	6,342	6,242	6,467	6,442	6,442	6,592	6,542	5,717
R-squared	0.2501	0.2603	0.2540	0.2527	0.2540	0.2517	0.2592	0.2381	0.2542	0.2503	0.2478	0.2518	0.2526	0.2640
Number of region	264	245	266	265	266	265	254	250	259	258	258	264	262	229
Mean of dependent variable	.42	.39	.42	.42	.42	.42	.43	.43	.41	.43	.42	.42	.42	.42

*** p < 0.01, ** p < 0.05, * p < 0.1. The table presents results for the estimation of the model in Eq. 1. Standard errors are in parentheses and clustered on the level of NUTS 2 regions. The sample start year is 1990 for all regressions. Control variables, region fixed effects, and country-year fixed effects are included in all regressions

**Table 9:** Drop ten and five year periods one-by-one

	(1)	(2)	(3)	(4)	(5)	(6)	(7)	(8)
VARIABLES	EIB Loan Dummy							
Work region dummy	0.1621***	0.1506***	0.1539***	0.1558***	0.1453***	0.1544***	0.1614***	0.1513***
	(0.0461)	(0.0470)	(0.0474)	(0.0474)	(0.0473)	(0.0492)	(0.0458)	(0.0487)
Without years	59-68	59-63	64-68	69-78	69-73	74-78	79-88	79-83
Observations	13,630	15,080	15,080	13,630	15,080	15,080	13,630	15,080
R-squared	0.3383	0.3599	0.3713	0.3790	0.3786	0.3873	0.3945	0.3898
Mean of dependent variable	.3	.27	.27	.28	.27	.26	.25	.26
Work region dummy	0.1551***	0.1613***	0.1536***	0.1496***	0.0871	0.1385***	0.1057*	0.1316*
	(0.0456)	(0.0406)	(0.0438)	(0.0458)	(0.0624)	(0.0496)	(0.0599)	(0.0698)
Without years	84-88	89-98	89-93	94-98	99-08	99-03	04-08	09-15
Observations	15,080	13,630	15,080	15,080	13,630	15,080	15,080	14,500
R-squared	0.3896	0.3785	0.3789	0.3868	0.4138	0.3953	0.4002	0.3779
Mean of dependent variable	.25	.23	.24	.24	.23	.24	.24	.22
Number of regions	290	290	290	290	290	290	290	290
Region FEs	×	×	×	×	×	×	×	×
Country-year FEs	×	×	×	×	×	×	×	×
Controls								

*** p < 0.01, ** p < 0.05, * p < 0.1. The table presents results for the estimation of the model in Eq. 1. Standard errors are in parentheses and clustered on the level of NUTS 2 regions

## Footnotes

1 To put this number into perspective, note that the EU’s annual budget, which does not include the EIB, is around twice this much (European Commission 2018).

2 For example, the EIB took a leading role in the European economic policy package to fight the effects of the Covid-19 crisis. Early in the crisis it was announced on April 9, 2020 that the EIB group would establish a European Guarantee Fund that shall mobilize 200 billion euros of finance for companies, which corresponds to 37% of the overall package (Eurogroup 2020). The global financial crisis presents another example of EIB’s role in anti-cyclical economic policy in Europe. See, e.g., Corsetti et al. (2020) for a discussion of how the EU utilized official lending to respond to the crisis.

3 See, e.g., Gehring and Schneider (2018) and Aksoy (2010) who show that, respectively, the EU Commissioner for Agriculture and the EU President are able to influence the budget allocation in favor of their home countries.

4 In additional tests we also study the Directors’ region of education.

5 We focus on direct project loans by the EIB. Projects that cost at least 25 million Euros qualify for this type of loans.

6 One exception in the literature documenting positive effects of home-bias in various outcomes is Fisman et al. (forthcoming) who shows that bureaucrats sharing a hometown or college connection with an incumbent member of the Chinese Politburo are actually penalized in their probability to be promoted to the Politburo.

7 Several papers try to isolate the favoritism and information channels in different contexts. For example, Khwaja and Mian (2005) find that politically connected firms in Pakistan borrow 45% more than their non-connected peers. By studying the outcomes of these loans they show that the connected firms have 50% higher default rates. Haselmann et al. (2018) show that German firms whose CEOs are in elite social networks with bank representatives receive more lending. Although the immediate terms of loans such as interest rates or defaults that connected firms receive are not different from those of control firms, the paper shows that the ex-post loan performance as measured by return on loans is substantially lower for connected firms. Unfortunately, data unavailability on the performance of EIB loans prohibits us from performing such an exercise. Another strategy is used by Fuchs and Gehring (2017). The paper first documents that rating agencies more favorably rate the sovereign bonds of their home countries. However, a test that compares countries where information is likely to be abundant to countries where information is not as easily accessible suggests that home biased ratings are probably not driven by the informational advantages of agencies towards their homes.

8 Nevertheless, there is evidence that the EU budget has positive albeit very small economic effects (Becker et al. 2010), which are only concentrated in areas with high levels of human capital and quality of government (Becker et al. 2013).

9 For a comparison between home-bias in intranational versus home-bias in international trade, see Wolf (2000).

10 A related field studies whether political connections of firms influence their opportunities to attract lending (among others, see Sapienza 2004; Khwaja and Mian 2005; Faccio et al. 2006; Haselmann et al. 2018).

11 Own calculations based on EIB (2020c)

12 In the 2017 Activity Report, the EIB describes some show-case examples: A project on innovation developed an in-organic substitute for coconut shells as supercapacitors. A microfinance firm in Luxembourg has been part of the support for SMEs. The expansion of electric cars in Paris was part of the infrastructure dimension and environmental projects included the rewilding of a region in Bulgaria (EIB 2017a).

13 With the departure from the European Union, the United Kingdom is not involved in the EIB anymore.

14 With the departure of the United Kingdom from the EU, Germany, France, and Italy now each hold 18.8% of the total shares (EIB 2020d).

15 To get an idea how the CVs look like, the official EIB website provides with the CVs of the current Board of Directors (EIB 2020e).

16 Countries are eligible for the Cohesion Fund in case their gross national income per inhabitant is lower than 90% of EU average. We use data from the European Commission (European Commission 2020) to look which regions actually received money from the Cohesion Fund.

17 The EIB provided us with 157 CVs. In these CVs, the Directors voluntarily list the prior workplaces and other information they wish to provide to the public. We then coded the regions manually from this information. For the remaining Directors, we took information on their work region from the EIB annual reports available in the Historical Archives of the European Union (European University Institute 2020). We also tried to complement the education region manually via a Google search, which increased the number of available education regions to 262.

18 These countries are Bulgaria, Czech Republic, Estonia, Latvia, Lithuania, Hungary, Poland, Romania, Slovenia and Slovakia.

19 See Section 2 for details.

20 The first column estimates our specification on the full sample starting in 1959. The sample for the rest of the table is restricted to the post-1990 period. This is the period when control variables are available for all regions. All regressions in Table 3 include region fixed effects. Columns 1 to 6 control for country-by-year effects, while the non-linear models of columns 7 and 8 instead control for year fixed effects. Column 4 additionally includes a region-specific linear time trend to account for the possibility that regions develop differently over time. In addition to controlling for contemporaneous GDP as part of our standrad set of control variable, Column 5 additionaly includes the moving 5-year average of GDP per capita in the 5 years proceeding the treatment year. This is done to make sure it is not the cofinancing of EU Structural and Investment Funds that drives our results.

21 Note that in these regressions, the country-fixed effects are left out due to computational reasons, and we instead introduce year fixed effects.

22 The treatment effects in Fig. 5 are larger than the baseline estimates of Table 3. One reason is that the figure shows the treatment on the individual Board member level and not on the region level as in the baseline regressions. For example, if a region is treated by two Board Directors who follow each others term, the dummy for the first year of treatment switches on for both of the Directors.

23 The point estimate in *t* − 1 is positive and larger than the one in *t* − 2, which might indicate a pre-trend, but it has a small size compared to the point estimates during tenure and is in any case statistically indistinguishable from zero.

24 These sectors are: Agriculture, Industry, Energy, Infrastructure, Non-market services, and Services.

25 In this matrix of 24 treatment estimates, we see one (three) more point estimates that are different from zero at the 5 (10%) level, which however do not show a meaningful pattern of effects on size within other industries.

26 The energy sector has a positive significant point estimate in column 6 which is not robust to the evidence in column 7. The agricultural sector has an implausible large significant effect in column 6 which is an artifact of having too few observations left after splitting the sample by sector size. As a result the estimation for agriculture in column 7 is not identified.

27 The CVs we have of the Board members do not cover the period after working at the EIB. Hence, we collected information on the workplace after the time at the EIB via internet search. In so doing, we find information on 132 Board members working at the EIB after 1990, who make around 40% of the sample of Directors over the same period. For the rest of the Directors, including those who served at the EIB before 1990, we fail to find reliable career information. Out of the 132 Director for whom we have data, 68 stayed in the same region and 64 switched to new regions, of which 51 are within the EU.

28 As explained in section 2, a large share of the work region variable was collected via annual reports of the EIB. This source does not provide with the years the Directors have already spent in one region. Therefore, we tried to complement the information of the CVs by manual Google search. In the end, we know the experience in one region for 202 Directors.

29 We do several tests to make sure that the positive effect for the work region and the absence for the education region is not driven by the poor availability of the education variable. In column 7, we first include both treatment effects together. In columns 8-9, we only include the Directors for which both the education and work region are available. The effects do not change.

## References

[CR1] Aksoy, D. (2010). Who gets what, when, and how revisited: voting and proposal powers in the allocation of the EU budget. *European Union Politics*, *11*(2), 171–194.

[CR2] Aksoy, D. (2012). Institutional arrangements and logrolling: evidence from the european union. *American Journal of Political Science*, *56*(3), 538–552.

[CR3] Alesina, A., Angeloni, I., & Schuknecht, L. (2005). What does the european union do? *Public Choice*, *123*(3-4), 275–319.

[CR4] Bachtler, J., & Mendez, C. (2007). Who governs EU cohesion policy? Deconstructing the reforms of the structural funds. *Journal of Common Market Studies*, *45*(3), 535–564.

[CR5] Baldwin, R.E., & Wyplosz, C. (2012). *The economics of european integration*. London: McGraw-Hill Higher Education.

[CR6] Barro, R.J., & Lee, J.-W. (2005). IMF programs: who is chosen and what are the effects? *Journal of Monetary Economics*, *52*, 1245–1269.

[CR7] Baskaran, T., & Lopes da Fonseca, M. (2018). Appointed public officials and local favoritsm: evidence from the German States. CESifo Working Paper Series No. 6800.

[CR8] Becker, S.O., Egger, P.H., & von Ehrlich, M. (2010). Going NUTS: the effect of EU structural funds on regional performance. *Journal of Public Economics*, *94*, 578–590.

[CR9] Becker, S.O., Egger, P.H., & von Ehrlich, M. (2013). Absorptive capacity and the growth and investment effects of regional transfers: a regression discontinuity design with heterogeneous treatment effects. *American Economic Journal: Economic Policy*, *5*(4), 29–77.

[CR10] Bodenstein, T., & Kemmerling, A. (2012). Ripples in a rising tide: why some EU regions receive more structural funds than others. *European Integration Online Papers (EIoP), 16*(1).

[CR11] Carozzi, F., & Repetto, L. (2016). Sending the pork home: birth town bias in transfers to Italian municipalities. *Journal of Public Economics*, *134*, 42–55.

[CR12] Carvalho, D. (2014). The real effects of government owned banks: Evidence from an emerging market. *The Journal of Finance*, *69*(2), 557–609.

[CR13] Chavaz, M., & Rose, A.K. (2019). Political borders and bank lending in post-crisis America. *Review of Finance*, *23*(5), 935–959.

[CR14] Clifton, J., Díaz-Fuentes, D., & Gómez, A.L. (2018). The European Investment Bank: development, integration, investment? *JCMS: Journal of Common Market Studies*, *56*(4), 733–750.

[CR15] Cole, S. (2009). Fixing market failures or fixing elections? Agricultural credit in India. *American Economic Journal: Applied Economics*, *1*(1), 219–250.

[CR16] Conley, T.G., & Taber, C.R. (2011). Inference with difference in differences with a small number of policy changes. *The Review of Economics and Statistics*, *93*(1), 113–125.

[CR17] Cornell, B., & Welch, I. (1996). Culture, information, and screening discrimination. *Journal of Political Economy*, *104*(3), 542–571.

[CR18] Corsetti, G., Erce, A., & Uy, T. (2020). Official sector lending during the euro crisis. *Review of International Organizations*.

[CR19] Counter Balance. (2016). Corrupt but legal - Institutionalised corruption and development finance. Counter Balance.

[CR20] Dahan, M. , & Yakir, I. (2019). Revealed political favoritism: evidence from the allocation of state lottery grants in Israel. CESifo Working Paper No. 7882.

[CR21] Dickens, A. (2018). Ethnolinguistic favoritism in African Politics. *American Economic Journal: Applied Economics*, *10*(3), 370–402.

[CR22] Dinc, I.S. (2005). Politicians and banks: Political influences on government-owned banks in emerging markets. *Journal of Financial Economics*, *77*(2), 453–479.

[CR23] Do, Q.-A., Nguyen, K.-T., & Tran, A.N. (2017). One mandarin benefits the whole clan: hometown favoritism in an authoritarian regime. *American Economic Journal: Applied Economics*, *9*(4), 1–29.

[CR24] Dreher, A., Fuchs, A., Hodler, R., Parks, B.C., Raschky, P.A., & Tierney, M.J. (2019). African leaders and the geography of China’s foreign assistance. *Journal of Development Economics*, *140*, 44 – 71.

[CR25] Dreher, A., & Lang, V.F. (2019). The political economy of international organizations. In *The Oxford handbook of public choice, vol 2, Chapter 31*. Oxford: Oxford University Press.

[CR26] Dreher, A., & Sturm, J.-E. (2012). Do the IMF and the World Bank influence voting in the UN General Assembly? *Public Choice*, *151*(1), 363–397.

[CR27] Dreher, A., Sturm, J.-E., & Vreeland, J.R. (2009). Global horse trading: IMF loans for votes in the United Nations. *European Economic Review*, *53*, 742–757.

[CR28] Dür, A., Moser, C., & Spilker, G. (2020). The political economy of the European Union. *Review of International Organizations*. 10.1007/s11558-020-09389-8 PMC7247867 38624314

[CR29] EIB. (2012). Code of Conduct for the Members of the Board of Directors. Technical report, European Investment Bank.

[CR30] EIB. (2015a). European investment bank - the governance. Technical report, European Investment Bank.

[CR31] EIB. (2015b). Operational plan 2015-2017. Technical report, European Investment Bank.

[CR32] EIB. (2017a). Activity report 2017. Technical report, European Investment Bank.

[CR33] EIB. (2017b). Financial report 2017. Technical report, European Investment Bank.

[CR34] EIB. (2018). Investment plan for Europe. Technical report, European Investment Bank.

[CR35] EIB. (2020a). Applying for a loan. https://www.eib.org/en/projects/cycle/applying_loan/index.htm.

[CR36] EIB. (2020b). Cohesion and regional development overview 2020. Technical report, European Investment Bank Group.

[CR37] EIB. (2020c). Financed projects data. http://www.eib.org/en/projects/loan/list/index.htm.

[CR38] EIB. (2020d). Shareholders. https://www.eib.org/en/about/governance-and-structure/shareholders/index.htm.

[CR39] EIB. (2020e). The board of directors. https://www.eib.org/en/about/governance-and-structure/statutory-bodies/board_of_directors/index.htm.

[CR40] Englmaier, F., & Stowasser, T. (2017). Electoral cycles in savings bank lending. *Journal of the European Economic Association*, *15*(2), 296–354.

[CR41] Eurogroup. (2020). Remarks by Mário Centeno following the Eurogroup video conference of 9 April 2020. https://www.consilium.europa.eu/en/press/press-releases/2020/04/09/remarks-by-mario-centeno-following-the-eurogroup-videoconference-of-9-april-2020/https://www.consilium.europa.eu/en/press/press-releases/2020/04/09/remarks-by-mario-centeno-following-the-eurogroup-videoconference-of-9-april-2020/.

[CR42] European Commission. (2018). Consolidated annual accounts of the European Union and financial statement – discussion and analysis. Technical report. Publications Office of the European Union.

[CR43] European Commission. (2020). European structural and investment funds data. https://cohesiondata.ec.europa.eu/. Accessed in 2018.

[CR44] European University Institute. (2020). Historical archives of the European Union: Banque européenne d’investissement. https://archives.eui.eu/en/fonds/30462?item=BEI.

[CR45] Fabre, B., & Sangnier, M. (2017). What motivates french pork: political career concerns or private connections? AMSE Working Paper 2017 Nr 5.

[CR46] Faccio, M., Masulis, R.W., & McConnell, J.J. (2006). Political connections and corporate bailouts. *The Journal of Finance*, *61*(6), 369–386.

[CR47] Fisman, R., Paravisini, D., & Vig, V. (2017). Cultural proximity and loan outcomes. *American Economic Review*, *107*(2), 457–92.

[CR48] Fisman, R., Shi, J., Wang, Y., & Wu, W. (forthcoming). Social ties and the selection of China’s political elite. *American Economic Review*.

[CR49] Fiva, J.H., & Halse, A.H. (2016). Local favoritism in at-large proportional representation systems. *Journal of Public Economics*, *143*, 15–26.

[CR50] Franck, R., & Rainer, I. (2012). Does the leader’s ethnicity matter? Ethnic favoritism, education and health in Sub-Saharan Africa. *American Political Science Review*, *106*, 294–325.

[CR51] Fuchs, A., & Gehring, K. (2017). The home bias in sovereign ratings. *Journal of the European Economic Association*, *15*(6), 1386–1423.

[CR52] Gehring, K., & Schneider, S.A. (2018). Towards the greater good? EU Commissioners’ Nationality and Budget Allocation in the European Union. *American Economic Journal: Economic Policy*, *10*(1), 214–39.

[CR53] Golden, M., & Min, B. (2013). Distributive politics around the world. *Annual Review of Political Science*, *16*, 73–99.

[CR54] Haselmann, R., Schoenherr, D., & Vig, V. (2018). Rent seeking in elite networks. *Journal of Political Economy*, *126*(4), 1638–1690.

[CR55] Hodler, R., & Raschky, P.A. (2014). Regional favoritism. *Quarterly Journal of Economics*, *129*, 995– 1033.

[CR56] Kaja, A. , & Werker, E. (2010). Corporate Governance at the World Bank and the Dilemma of Global Governance. *World Bank Economic Review*, *24*(2), 171–198.

[CR57] Khwaja, A.I., & Mian, A. (2005). Do lenders favor politically connected firms? Rent provision in an emerging financial market. *Quarterly Journal of Economics*, *120* (4), 1371–1411.

[CR58] Kilby, C. (2009). The political economy of conditionality: an empirical analysis of World Bank loan disbursements. *Journal of Development Economics*, *89*, 51–61.

[CR59] Kramon, E., & Posner, D.N. (2013). Who benefits from distributive politics? How the outcome one studies affects the answer one gets. *Perspectives on Politics*, *11*, 461–474.

[CR60] Kramon, E., & Posner, D.N. (2016). Ethnic favoritism in primary education in Kenya. *Quarterly Journal of Political Science*, *11*, 1–58.

[CR61] Kudamatsu, M. (2007). Ethnic favoritism: micro evidence from Guinea. Mimeo (University of Stockholm).

[CR62] Mazzucato, M., & Penna, C.C. (2016). Beyond market failures: the market creating and shaping roles of state investment banks. *Journal of Economic Policy Reform*, *19*(4), 305–326.

[CR63] Mertens, D., & Thiemann, M. (2019). Building a hidden investment state? The European Investment Bank, national development banks and European economic governance. *Journal of European Public Policy*, *26*(1), 23–43.

[CR64] Mikulaschek, C. (2018). Issue linkage across international organizations: does European countries’ temporary membership in the UN Security Council increase their receipts from the EU budget? *The Review of International Organizations*, *13*(4), 491–518.

[CR65] Miquel, G.P.I. (2007). The control of politicians in divided societies: the politics of fear. *Review of Economic Studies*, *74*, 1259–1274.

[CR66] Moser, C., & Sturm, J.-E. (2011). Explaining IMF lending decisions after the Cold War. *The Review of International Organizations*, *6*(3-4), 304–340.

[CR67] Persson, P., & Zhuravskaya, E. (2016). The limits of career concerns in federalism: evidence from China. *Journal of the European Economic Association*, *14*(2), 338–374.

[CR68] Rajan, R.G. (1992). Insiders and outsiders: the choice between informed and arm’s-length debt. *The Journal of Finance*, *47*(4), 1367–1400.

[CR69] Robinson, N. (2009). The European investment Bank: the EU’s Neglected Institution. *Journal of Common Market Studies*, *47*(3), 651–673.

[CR70] Sapienza, P. (2004). The effects of government ownership on bank lending. *Journal of Financial Economics*, *72*(2), 357–384.

[CR71] Schneider, C.J. (2013). Globalizing electoral politics: political competence and distributional bargaining in the European Union. *World Politics*, *65*(3), 452–490.

[CR72] Stiglitz, J.E. (1994). The role of the state in financial markets. In *Proceedings of the World Bank annual conference on economic development 1993* (pp. 19–52).

[CR73] Stone, R.W. (2004). The political economy of IMF Lending in Africa. *American Political Science Review*, *98*(4), 577–591.

[CR74] Sturm, J.-E., & de Haan, J. (2005). Which variables explain decisions on IMF credit? An extreme bounds analysis. *Economics and Politics*, *17*, 177–213.

[CR75] Transparency International EU. (2016). Investing in integrity? Transparency and accountability of the European Investment Bank. Transparency International EU.

[CR76] Wolf, H.C. (2000). Intranational home bias in trade. *Review of Economics and Statistics*, *82*(4), 555–563.

