# Supplementary material for: The political economy of multilateral lending to European regions
Source: Rev Int Organ. 2020 Jul 2;15(3):707–40. doi: 10.1007/s11558-020-09385-y (PMC7330003; doi:10.1007/s11558-020-09385-y)
Supplement: Supplementary file 1 — (ZIP 10.4 MB) [file 11558_2020_9385_MOESM1_ESM.zip › Public Data April20/AsatryanHavlik2020_PublicData.pdf]

ONLINE APPENDIX:

The Political Economy of Multilateral Lending to European Regions

Zareh Asatryan*† Annika Havlik*‡

*ZEW Mannheim †CESifo ‡University of Mannheim

April 27, 2020

- We use three sets of data:
  - Project-level data on EIB loans (N=15,932)[1]: includes the size of loans, time of commitment, region (i.e., NUTS) identifier, name of project, sector, etc.[2]
  - Person-level data on EIB Board of Directors (N=470): regions of workplace for all 470 Directors[3] and regions of education of highest degree for 262 Directors.[4]
  - Person-level data on EIB Management Committee (N=70): regions of workplace for 49 Directors[5]
  - Region-level data on socio-economic characteristics: includes GDP, population, compensation and hours worked per employee, and gross fixed capital

* *E-mail addresses:* zareh.asatryan@zew.de, annika.havlik@zew.de

[1]This is the total number of loans in the data, flowing to all different levels of regions. See also Table 1 in the manuscript for details.

[2]The source of this data is the EIB.

[3]The work region was hand-collected from 157 CVs provided by the EIB, and for the remaining 313 Directors it was complemented with data collected from EIB annual reports available in the Historical Archives of the European Union (European University Institute 2020). We have full coverage for this variable.

[4]The education region was likewise hand-collected via the CVs and augmented via a manual Google search. Here, we have a partial coverage of only 262 Directors.

[5]The work region was equally hand-collected CVs provided by the EIB.

formation;[6] as well as data on disbursement of European Structural and Investment Funds (European Commission 2020) and data on distances between regions (Eurostat 2020).

- We aggregate our data to the level of European regions:
  - We use the NUTS 2 level of aggregation (classification as of 2010) for our main analysis.[7]
  - This gives us 291 regions in total.[8]
  - The sample starts in 1959 and ends in 2015.
  - In total, we have a balanced sample of 16,530 observations.
  - For summary statistics see Table 1 below.
  - For robustness tests we also use data aggregated to NUTS1, NUTS3 and NUTS2-quarterly level (see do files for further details).
- Do files:
  - The do file Asatryan_Havlik_dataprep.do, first, aggregates the raw project- and person-level data into region-level data, and merges all necessary variables.
  - The do file Asatryan_Havlik_descriptives.do plots all the descriptive figures and tables of the paper.
  - The do file Asatryan_Havlik_regressions.do estimates all the regressions of the paper.

[6]This database is called the European Regional Database and it was purchased from Cambridge Economics.

[7]The countries Estonia, Latvia, and Lithuania are exceptions where we use the NUTS 3 aggregation.

[8]Brussels region will we dropped from the analysis (NUTS code BE10) as explained in Section 2 of the manuscript.

Table 1: Summary statistics of variables in public data

Variable	Label	Obs	Mean	Std.Dev.	Min	Max
year	year	16,530	1987	16.45	1959	2015
nuts_code	NUTS code, string					
nuts_code_panel	NUTS code for panel ID	16,530	145.5	83.72	1	290
country_code	Country code, string					
country_code_panel	Country code for panel ID	16,530	14.64	8.408	1	28
analysis	Region-time level used for analysis					
first_year_work_full	First year at EIB, work region, Full member	16,530	0.0126	0.111	0	1
last_year_work_full	Last year at EIB, work region, Full member	16,530	0.0128	0.113	0	1
max_office_work_full	Number of years at EIB, work region, Full member, maximum for several people	730	5.421	3.978	1	22
first_year_work_alt	First year at EIB, work region, Alternate member	16,530	0.00938	0.0964	0	1
last_year_work_alt	Last year at EIB, work region, Alternate member	16,530	0.00980	0.0985	0	1
max_office_work_alt	Number of years at EIB, work region, Alt. member, maximum for several people	468	4.737	3.621	1	21
first_year_educ_full	First year at EIB, educ. region, Full member	16,530	0.00901	0.0945	0	1
last_year_educ_full	Last year at EIB, educ. region, Full member	16,530	0.00907	0.0948	0	1
max_office_educ_full	Number of years at EIB, educ. region, Full member, maximum for several people	597	4.363	3.351	1	19
first_year_educ_alt	First year at EIB, educ. region, Alternate member	16,530	0.00581	0.0760	0	1
last_year_educ_alt	Last year at EIB, educ. region, Alternate member	16,530	0.00551	0.0740	0	1
max_office_educ_alt	Number of years at EIB, educ. region, Alt. member, maximum for several people	276	2.866	1.932	1	13
max_office_work	Number of years at EIB, work region, maximum for several people	813	5.748	4.070	1	22
max_office_educ	Number of years at EIB, educ. region, maximum for several people	751	4.185	3.163	1	19
first_year_work	First year at EIB, work region	16,530	0.0180	0.133	0	1
last_year_work	Last year at EIB, work region	16,530	0.0178	0.132	0	1
first_year_educ	First year at EIB, education region	16,530	0.0137	0.116	0	1
last_year_educ	Last year at EIB, education region	16,530	0.0130	0.113	0	1
max_reg_start_work	Number of years in region when starting at EIB, max. for several people	16,530	0.145	1.458	0	33.36
treated_work	Work region dummy	16,530	0.0492	0.216	0	1
treated_educ	Education region dummy	16,530	0.0454	0.208	0	1
cat_exp	Number of years in region when starting at EIB, in 3-year intervals	16,530	0.0474	0.394	0	5

Table 1: Summary statistics of variables in public data (continued)

Variable	Label	Obs	Mean	Std.Dev.	Min	Max
treated_work_reduced	Work region dummy with both regions known	16,530	0.0319	0.176	0	1
treated_educ_reduced	Education region dummy with both regions known	16,530	0.0454	0.208	0	1
first_year_old	First year at EIB, pre-EIB region	16,530	0.00278	0.0527	0	1
last_year_old	Last year at EIB, pre-EIB region	16,530	0.00272	0.0521	0	1
max_office_old	Number of years at EIB, pre-EIB region, maximum for several people	152	3.303	1.993	1	10
first_year_post	First year at EIB, post-EIB region	16,530	0.00200	0.0446	0	1
last_year_post	Last year at EIB, post-EIB region	16,530	0.00206	0.0453	0	1
max_office_post	Number of years at EIB, post-EIB region, maximum for several people	115	3.504	2.075	1	10
MC_first_year_work	First year at EIB, Management Committee, work region	16,530	0.00248	0.0497	0	1
MC_last_year_work	Last year at EIB, Management Committee, work region	16,530	0.00194	0.0440	0	1
max_mc_office_work	Number of years at EIB, Management Committee, maximum for several people	235	5.591	4.681	1	24
MC_first_year_educ	First year at EIB, Management Committee, education region	16,530	0.00212	0.0460	0	1
MC_last_year_educ	Last year at EIB, Management Committee, education region	16,530	0.00157	0.0396	0	1
max_mc_office_educ	Number of years at EIB, Management Committee, maximum for several people	186	4.726	3.740	1	19
sum_loans	EIB loans in million euros	16,530	28.70	99.26	0	1874
iqr_loans	Interquartile range of loans per region-year	4,148	2.550e+07	5.860e+07	0	9.850e+08
sd_loans	Standard deviation of loans per region-year	2,418	3.220e+07	4.890e+07	0	6.970e+08
gdp	Billions of euros, 2005 constant prices, ERD	9,251	35.57	45.27	0.271	565.0
population	Thousands of people, ERD	9,249	1694	1477	22.76	12070
gva	Gross value added, millions of euros in 2005 constant prices, ERD	9,251	31915	40913	99.52	511771

Table 1: Summary statistics of variables in public data (continued)

Variable	Label	Obs	Mean	Std.Dev.	Min	Max
active_population	Employed+unemployed pop. minus students+pensioners, living place measure, ERD	8,723	842.7	710.6	8.169	6008
employment	Thousands of people, workplace measure, ERD	9,251	734.5	666.2	11.03	6115
hours_worked	Total annual hours worked, in million, ERD	8,686	1310	1150	19.43	9572
compensation_employees	Total renumeration of employees, million euros in 2005 prices, ERD	8,726	18236	21533	219.6	246062
gross_fixed_capital_formation	Million euros in 2005 prices, ERD	8,736	7763	9279	46.97	124611
ln_population	Ln population	9,249	13.98	0.927	10.03	16.31
compensation_employees_1000pc	Compensation per employee, in 2005 constant 1000 euros	8,726	22.59	10.64	1.162	105.7
hours1000_worked_pc	1000 hours worked per employee	8,686	1.718	0.204	1.293	2.456
ln_gfcf	Ln gross fixed capital formation	8,736	22.26	1.102	17.66	25.55
ln_gdp	Ln GDP	9,251	23.67	1.251	19.42	27.06
gdp_mio	GDP, millions of euros, 2005 constant prices	9,251	35569	45274	271	564982
gdp_pc	GDP per capita, 2005 constant prices	9,249	19435	10442	1585	98787
gdp_pc_mov_avg_5yr	GDP p.c. from t-6 to t-1	8,960	18822	10118	1708	96309
ln_gdp_pc_movavg_5yr	Ln GDP p.c. from t-6 to t-1	8,960	9.652	0.698	7.443	11.48
ln_sum_eu_payments	Ln sum of European Structural and Investment Funds	16,530	6.474	8.479	0	21.60
capital_city	capital	16,530	0.100	0.300	0	1
year_joining_EU	year_joining_EU	1,653	1989	17.81	1959	2013
capital_joinEU	Value=1 if nuts region is capital city and after joining EU	16,530	0.0457	0.209	0	1
loan_dummy	EIB loan dummy	16,530	0.251	0.434	0	1
share_loans	Share of regional loans in total loans in given year	16,530	0.00345	0.0148	0	0.471
ln_loans_nozeros	Ln EIB loans without zeros	4,148	3.797	1.546	-2.717	7.536
share_loans_gdp	Share of loans in GDP	9,251	0.00139	0.00397	0	0.0733
spl_treated_work	Spatial lag (dist) EIB directors	16,473	0.0525	0.0298	0.0132	0.198

References

European Commission (2020). European Structural and Investment Funds Data. https://cohesiondata.ec.europa.eu/ [Accessed in 2018].

European University Institute (2020). Historical Archives of the European Union: Banque européenne d'investissement. https://archives.eui.eu/en/fonds/30462?item=BEI [Accessed in 2016].

Eurostat (2020). Distances between NUTS regions. https://ec.europa.eu/eurostat/tercet/flatfiles.do [Accessed in 2018].
